# Supplementary figures and images for: PanoView: An iterative clustering method for single-cell RNA sequencing data
Source: PLoS Comput Biol. 2019 Aug 30;15(8):e1007040. doi: 10.1371/journal.pcbi.1007040 (PMC6742414; doi:10.1371/journal.pcbi.1007040)

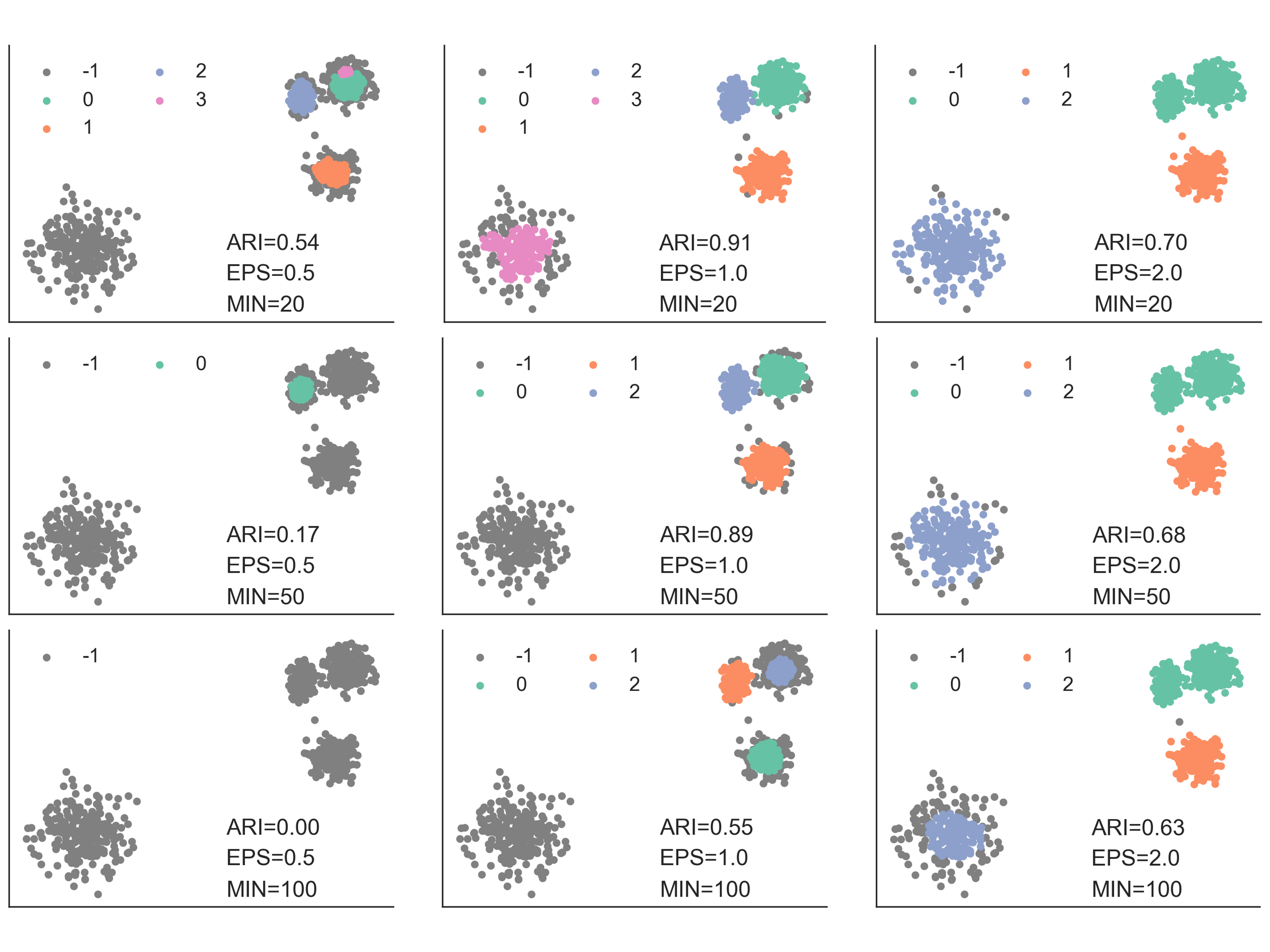

Supplement: S1 Fig — Clustering results of DBSCAN with different sets of parameters (epsilon and minPts). ARI value represents the similarity between the DBSCAN result and the ground truth. The value of 1 would indicate the clustering membership is the same as the ground truth. (TIF) [file pcbi.1007040.s001.TIF]

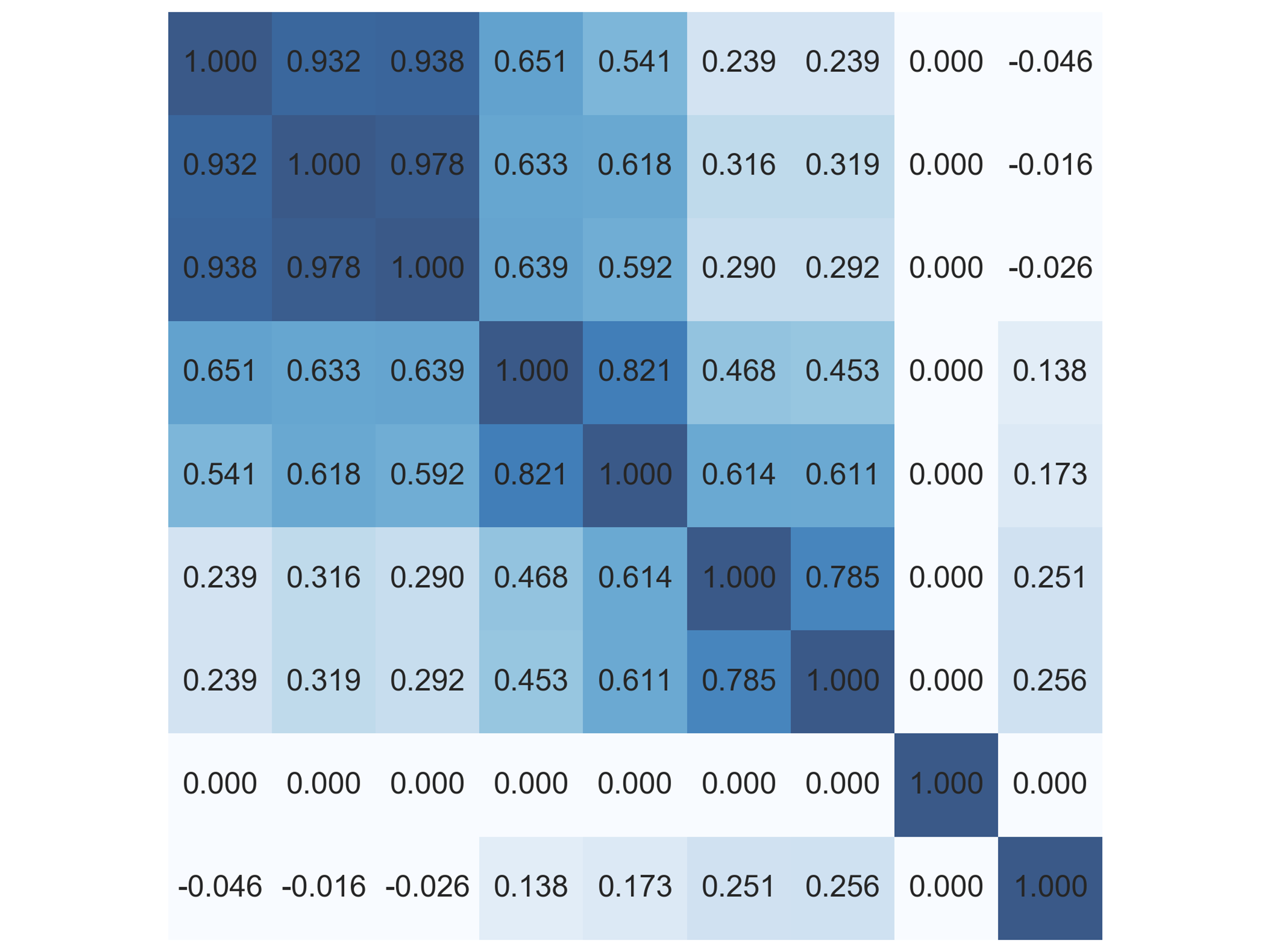

Supplement: S2 Fig — The pairwise comparison of clustering results from S1 Fig. Each value represents the ARI of the results from two different parameter sets. (TIF) [file pcbi.1007040.s002.TIF]

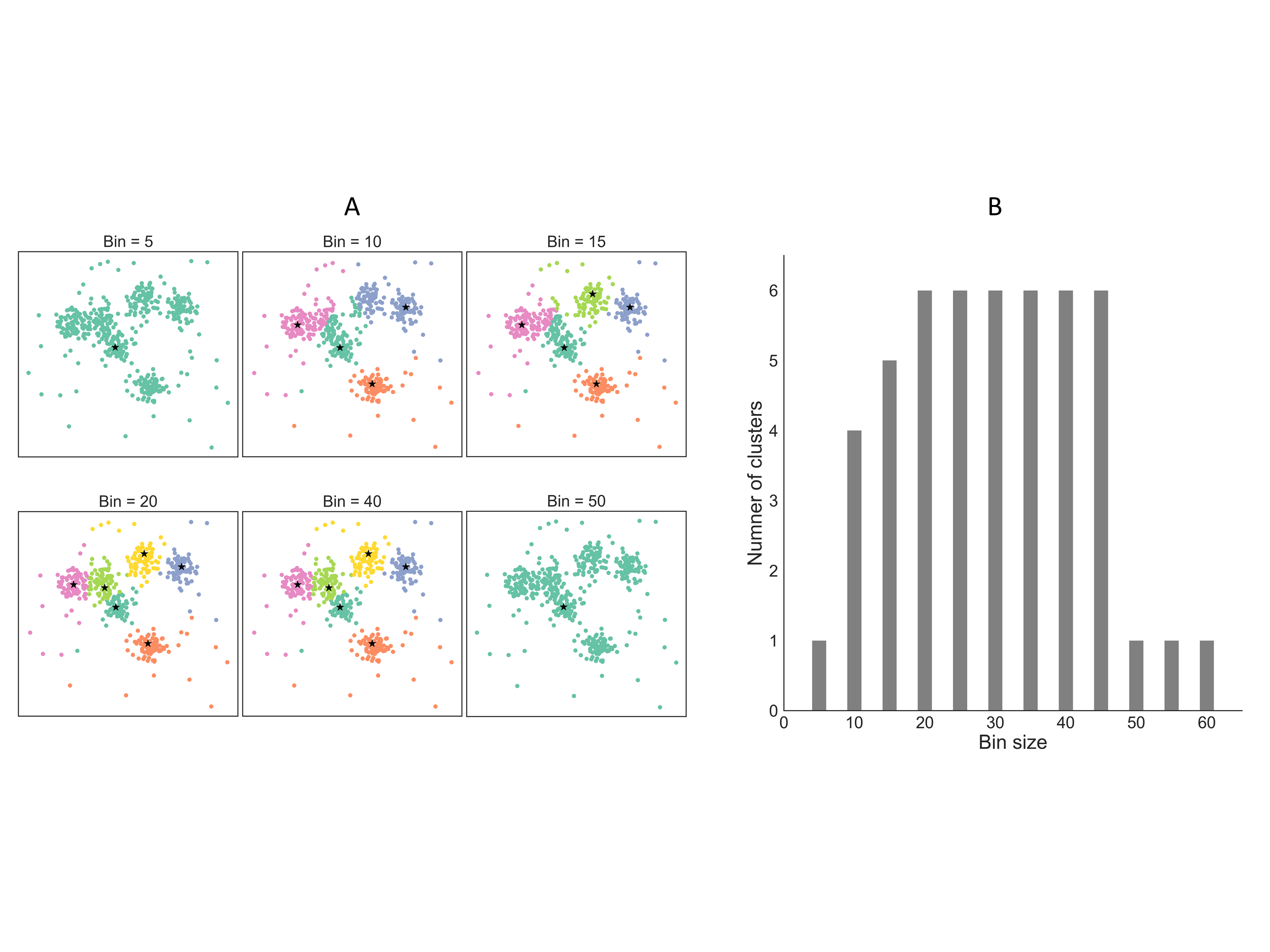

Supplement: S3 Fig — (A) The result of OLMC on 500 random 2D points analyzed using different bin sizes. (B): Optimal bin size is between 20 to 45 for this simulated data. (TIF) [file pcbi.1007040.s003.TIF]

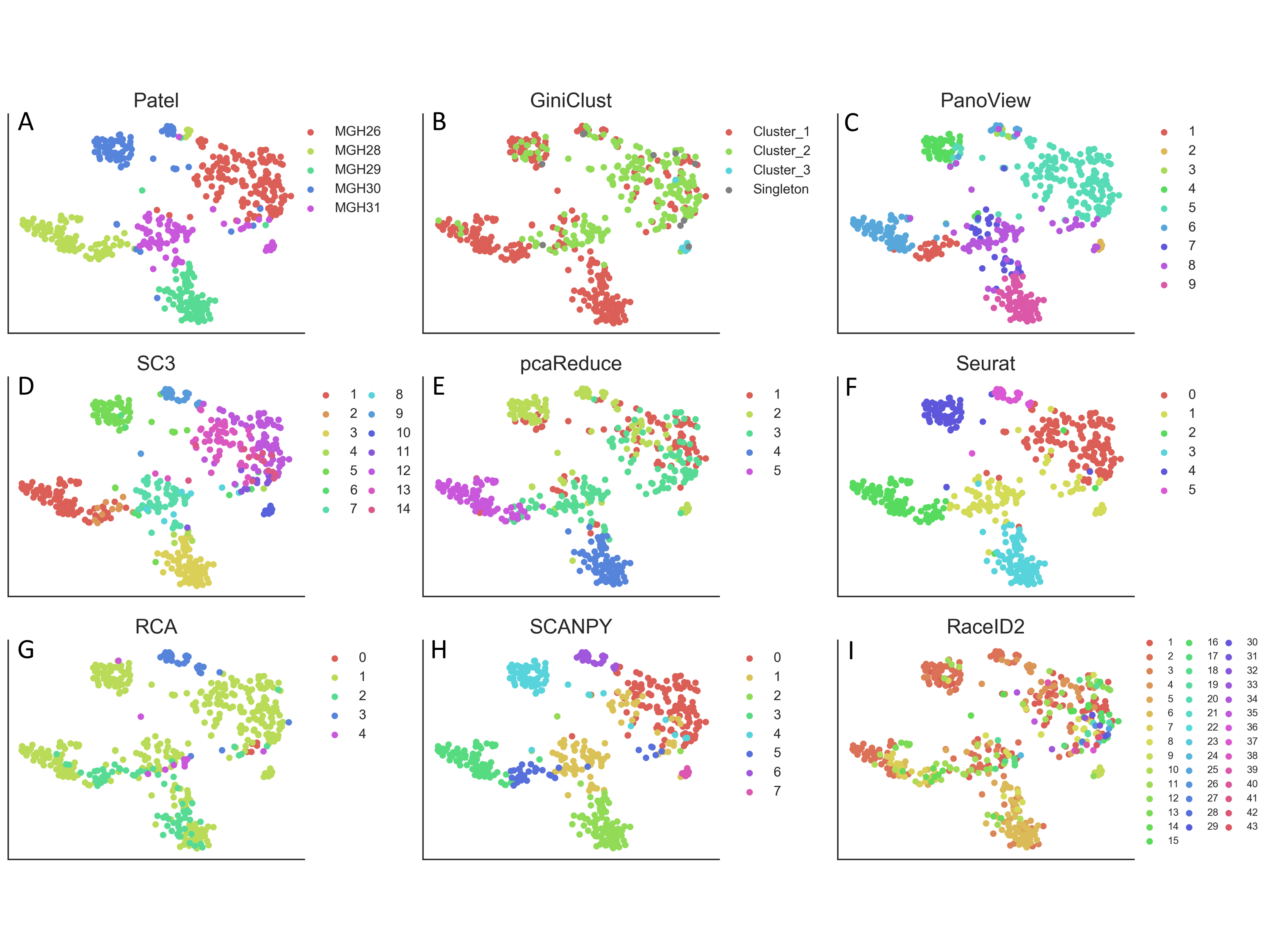

Supplement: S4 Fig — Comparison of different clustering methods using the Patel dataset. Visualization of clusters was generated by t-SNE. Panel A shows the original clustering results from the Patel et al publication. Panels B to I show the clustering results with different methods. (TIF) [file pcbi.1007040.s004.TIF]

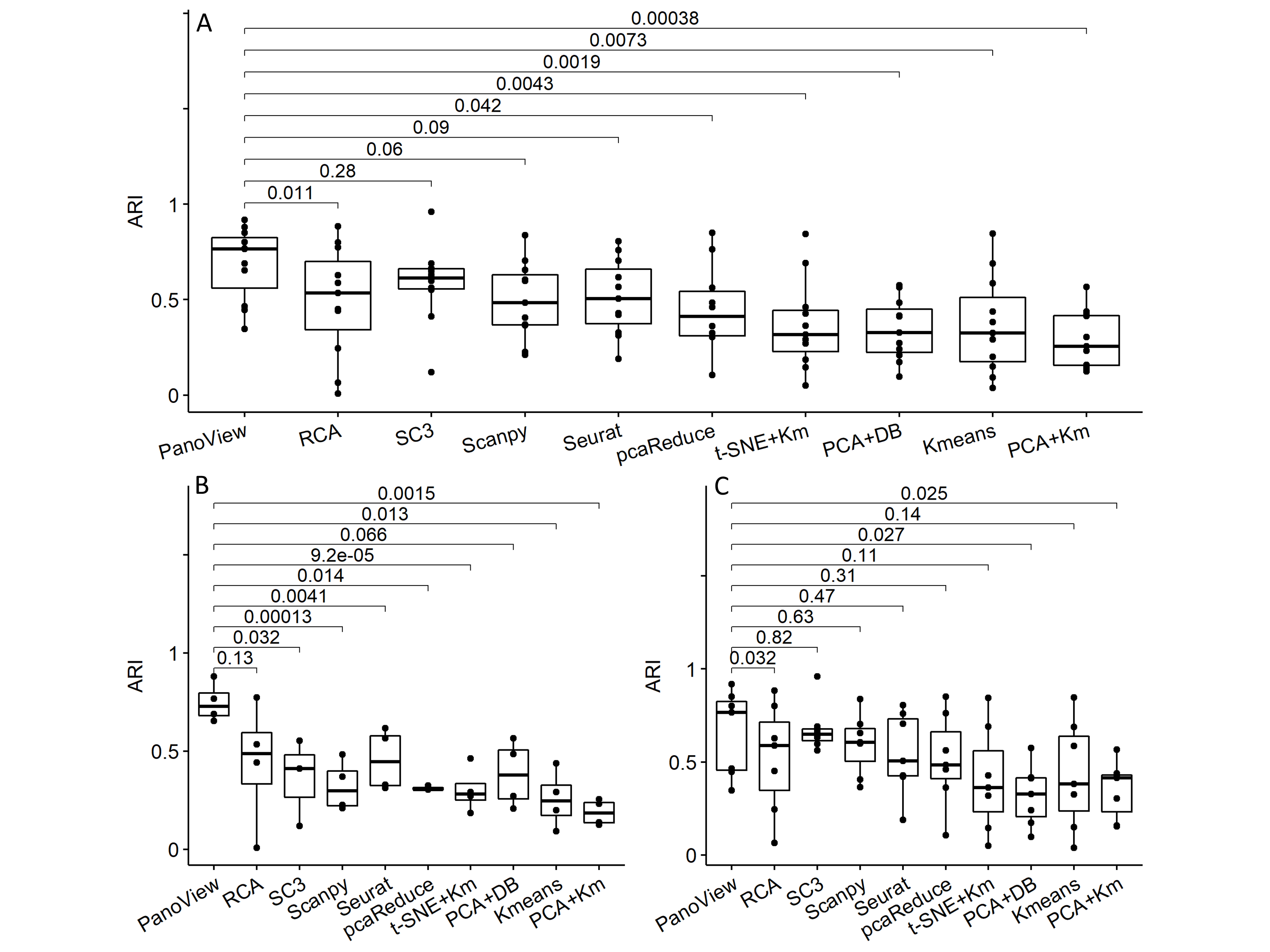

Supplement: S5 Fig — (A) The result of t-test between PanoView and other methods in 11 scRNA-seq datasets. Values in the figure are p-values that indicate the significant difference between two methods. (B) The result of statistical t-test in the datasets that contain more than 3,000 cells. (C): The result of the statistical test in the datasets that contain fewer than 3,000 cells. (TIF) [file pcbi.1007040.s005.tif]
