# Supplementary material for: PanoView: An iterative clustering method for single-cell RNA sequencing data
Source: PLoS Comput Biol. 2019 Aug 30;15(8):e1007040. doi: 10.1371/journal.pcbi.1007040 (PMC6742414; doi:10.1371/journal.pcbi.1007040)
Supplement: S1 Table — Key parameters for some computational methods used in scRNA-seq (DOCX) [file pcbi.1007040.s006.docx]

Table S1: **Key parameters in some computational methods for scRNA-seq**. Key parameters for some computational methods used in scRNA-seq

| **Methods** | **Parameters** | **Note** | **Stochastic** |
| --- | --- | --- | --- |
| PCA | N | number of components | No |
| tSNE | perplexity | number of nearest neighbors | Yes |
|  | early_exaggeration | how tight natural clusters are |  |
| Kmeans | K | number of clusters | No |
| DBSCAN | eps | distance to find neighboring points | No |
|  | min_points | number of points in the cluster |  |
| SC3 | ks | range of number of clusters | Yes |
|  | k_est | recommend number of clusters |  |
| pcaReduce | nbt | number of executions | Yes |
|  | q | range of number of clusters |  |
| Seurat | x.low/high.cutoff, y.low/high.cutoff | cutoff for variable genes | No |
|  | pcs.compute, dim.use | pca components for clustering |  |
| SCC-Cliq | k | neighboring distance | No |
|  | r | quasi-clique finding |  |
|  | m | distance for merging clusters |  |
| GiniClust | p_value_cutoff | cutoff for how many genes used | No |
|  | eps, min_points | same as DBSCAN |  |
| SCANPY | min_mean, max_mean, min_disp | Thresholds for variable genes | No |
|  | n_neighbors | number of neighboring cells |  |
|  | n_pcs | number of PCA components |  |
| RaceID2 | clustnr | number of cluster for gap statistic | No |
|  | outminc | expression cutoff |  |
|  | outlg | number of genes for an outlier |  |
|  | outdistquant | similarity cutoff for merging outliers |  |
|  | thr | probability for calculating outliers |  |
