# Supplementary material for: PanoView: An iterative clustering method for single-cell RNA sequencing data
Source: PLoS Comput Biol. 2019 Aug 30;15(8):e1007040. doi: 10.1371/journal.pcbi.1007040 (PMC6742414; doi:10.1371/journal.pcbi.1007040)
Supplement: S2 Table — Published scRNA-seq datasets used in this study. N is the total number of cells. K is the reported number of clusters in the original published studies (DOCX) [file pcbi.1007040.s007.docx]

Table S2: **scRNA-seq datasets used in this study**. Published scRNA-seq datasets used in this study. N is the total number of cells. K is the reported number of clusters in the original published studies.

| **Dataset** | **N** | **K** | **Units** |
| --- | --- | --- | --- |
| Campbell | 20,921 | 20 | UMI |
| Baron | 8569 | 14 | UMI |
| Tirosh | 4645 | 8 | TPM |
| Zeisel | 3005 | 9 | UMI |
| Villani | 1078 | 10 | TPM |
| Usoskin | 622 | 11 | RPM |
| Patel | 430 | 5 | TPM |
| Pollen | 301 | 11 | TPM |
| Deng | 268 | 10 | RPKM |
| Goolam | 124 | 5 | CPM |
| Yan | 90 | 7 | RPKM |
